# SNF1-Related Protein Kinases SnRK2.4 and SnRK2.10 Modulate ROS Homeostasis in Plant Response to Salt Stress

**DOI:** 10.3390/ijms20010143

**Published:** 2019-01-02

**Authors:** Katarzyna Patrycja Szymańska, Lidia Polkowska-Kowalczyk, Małgorzata Lichocka, Justyna Maszkowska, Grażyna Dobrowolska

**Affiliations:** Institute of Biochemistry and Biophysics, Polish Academy of Sciences, Pawińskiego 5a, 02-106 Warsaw, Poland; lidekp@ibb.waw.pl (L.P.-K.); mlichocka@ibb.waw.pl (M.L.); j.maszkowska@ibb.waw.pl (J.M.)

**Keywords:** antioxidant enzymes, *Arabidopsis thaliana*, ascorbate cycle, hydrogen peroxide, reactive oxygen species, salinity, SnRK2

## Abstract

In response to salinity and various other environmental stresses, plants accumulate reactive oxygen species (ROS). The ROS produced at very early stages of the stress response act as signaling molecules activating defense mechanisms, whereas those produced at later stages in an uncontrolled way are detrimental to plant cells by damaging lipids, DNA, and proteins. Multiple systems are involved in ROS generation and also in ROS scavenging. Their level and activity are tightly controlled to ensure ROS homeostasis and protect the plant against the negative effects of the environment. The signaling pathways responsible for maintaining ROS homeostasis in abiotic stress conditions remain largely unknown. Here, we show that in *Arabidopsis thaliana*, two abscisic acid- (ABA)-non-activated SNF1-releted protein kinases 2 (SnRK2) kinases, SnRK2.4 and SnRK2.10, are involved in the regulation of ROS homeostasis in response to salinity. They regulate the expression of several genes responsible for ROS generation at early stages of the stress response as well as those responsible for their removal. Moreover, the SnRK2.4 regulate catalase levels and its activity and the level of ascorbate in seedlings exposed to salt stress.

## 1. Introduction

Plants growing in nature are exposed to ever changing environmental conditions. They experience various abiotic stresses, such as drought, temperature extremes, and salinity. Salinity and drought are among the most detrimental factors limiting plant growth and development. Salinity causes ion-related stress, limitations in nutrient uptake as well as osmotic stress.

A secondary effect of salt stress and several other stresses is the accumulation of reactive oxygen species (ROS) in plant cells. Various ROS, such as singlet oxygen (^1^O_2_), superoxide radical (O_2_^−^), hydroxyl radical (−OH), and hydrogen peroxide (H_2_O_2_), are produced at low levels in chloroplasts, mitochondria, peroxisomes, and the apoplast during plant growth in optimal conditions [1,2]. They are involved in the regulation of plant growth and development, acting as signaling molecules. Upon stress, however, ROS play a double role [1,3,4,5]. ROS production at a low level is needed at the first stages of the stress response for induction of the plant defense, e.g., activation of signaling cascades, expression of stress response genes encoding enzymes involved in the synthesis of osmoprotectants, and some enzymes responsible for ROS scavenging [6,7,8]. At the later stages, ROS that accumulate in a non-controlled way have a widespread toxic effect, causing peroxidation of lipids, and damaging proteins and DNA, eventually leading to cell death. In response to stress, ROS are produced by diverse enzymes, e.g., NADPH oxidases, glycolate oxidases, oxalate oxidase, xanthine oxidase, and some peroxidases [9,10]. In *Arabidopsis thaliana* subjected to salinity stress, mainly two NADPH oxidases, respiratory burst oxidases, AtRbohD and AtRbohF, are involved in ROS production. They generate O_2_^−^ free radicals in the apoplastic space by transferring electrons from NADPH to O_2_. Then, the O_2_^−^ is dismutated to H_2_O_2_ by superoxide dismutase (SOD) and the H_2_O_2_ molecules diffuse to adjacent cells, where they can play a role of signaling molecules, inducing plant defense, or they cause oxidative stress and cell damage. To achieve ROS homeostasis, which is required for efficient defense against the negative effects of harmful environmental conditions, plants have evolved several systems for ROS removal, both enzymatic, such as catalases (CATs), SODs, and various peroxidases (PRXs), and non-enzymatic, i.e., the ascorbate-glutathione cycle [9,11,12,13,14,15,16]. The enzymes involved in the ROS production and removal are encoded by multiple genes and are strictly regulated in response to stress, at the transcriptional, protein, and activity levels.

There are several reports showing that kinases from the SNF1-releted protein kinases 2 (SnRK2s) family are major regulators of the plant response to osmotic stress (drought, salinity). SnRK2s are plant-specific kinases activated in response to osmotic stress and some of them additionally in response to abscisic acid (ABA). They have been found in every plant species analyzed [17]. The SnRK2s are classified into three groups based on their phylogeny. The classification correlates well with their response to ABA: Group 1 comprises ABA-non-activated kinases; in group 2, are kinases weakly activated or non-activated by ABA (depending on plant species); and group 3 contains kinases strongly activated by ABA [18,19]. SnRK2s play a crucial role in the induction of defense mechanisms against drought [20,21] and salinity [22,23,24] via ABA-dependent and ABA-independent pathways. So far, the role of ABA-dependent SnRK2s (SnRK2.2, SnRK2.3, and SnRK2.6) has been mostly studied and found to be crucial for ABA signaling [25,26]. In response to drought, they regulate stomatal closure in an ABA-dependent manner by phosphorylating several ion channels [27,28,29], expression of stress-response genes by phosphorylating transcription factors activated in response to ABA [30,31,32], and the activity of aquaporins [33]. SnRK2.6 is involved in the ABA-dependent ROS production indispensable for stomatal closure [34], possibly by phosphorylating the NADPH oxidase, RbohF [35,36].

Much less is known about the involvement of SnRK2s in response to salinity, even though these kinases are known to be strongly activated in response to this stress. It has been shown that ABA-non-responsive kinases (belonging to group 1) are activated rapidly and transiently in response to salt stress [23,37,38]. McLoughlin et al. [23] showed that two kinases from group 1, SnRK2.4 and SnRK2.10, are fully active within seconds in roots of Arabidopsis plants after treatment with NaCl. Both kinases were found to be required for plant tolerance to salinity stress by regulating root growth and architecture [23]. Moreover, it has been shown that SnRK2s from group 1 influence the plant tolerance to salt stress via regulation of mRNA decay. They phosphorylate VARICOSE (VCS), a protein regulating mRNA decapping [24]. Very recently, using the phosphoproteomic approach, several potential ABA-non-activated SnRK2s’ targets that phosphorylated in response to salinity have been found [39]. Among them there were several proteins, e.g., RNA- and DNA- binding proteins, protein kinases, phosphatases, and dehydrins, Early Responsive to Dehydration 10 (ERD10) and ERD14, whose phosphorylation likely affects the plant tolerance to salt stress. It has also been suggested that kinases from this group could be involved in the regulation of tolerance to salt stress via regulation of oxidative stress generated in response to salinity. Diédhiou et al. [22] showed that transgenic rice overexpressing Stress-Activated Protein Kinase 4 (SAPK4), the rice ABA-non-activated SnRK2, exhibited improved tolerance to salt stress. Their results indicated that SAPK4 regulates Na^+^ and Cl^−^ accumulation and ROS homeostasis; the enhanced level of the kinase caused up-regulation of the *CatA* gene encoding catalase. Additionally, it has been shown that SnRK2.4 positively regulates the accumulation of ROS in response to stress induced by cadmium ions [40].

These data suggest that at least some members of the group 1 of the SnRK2 family are likely to be involved in the regulation of plant tolerance to osmotic stress by controlling the ROS level. The aim of the present study was to establish the role of SnRK2.4 and SnRK2.10 in the regulation of the ROS homeostasis in response to salt stress in *Arabidopsis thaliana*.

## 2. Results

### 2.1. SnRK2.4 and SnRK2.10 Kinases Are Involved in H_2_O_2_ Accumulation in Response to Salt Stress

**Figure 1:**
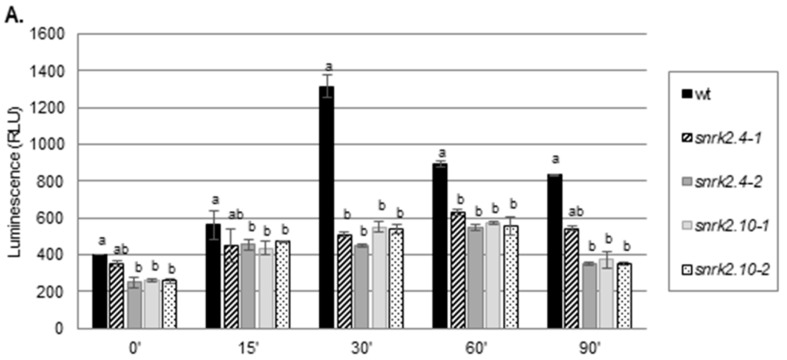

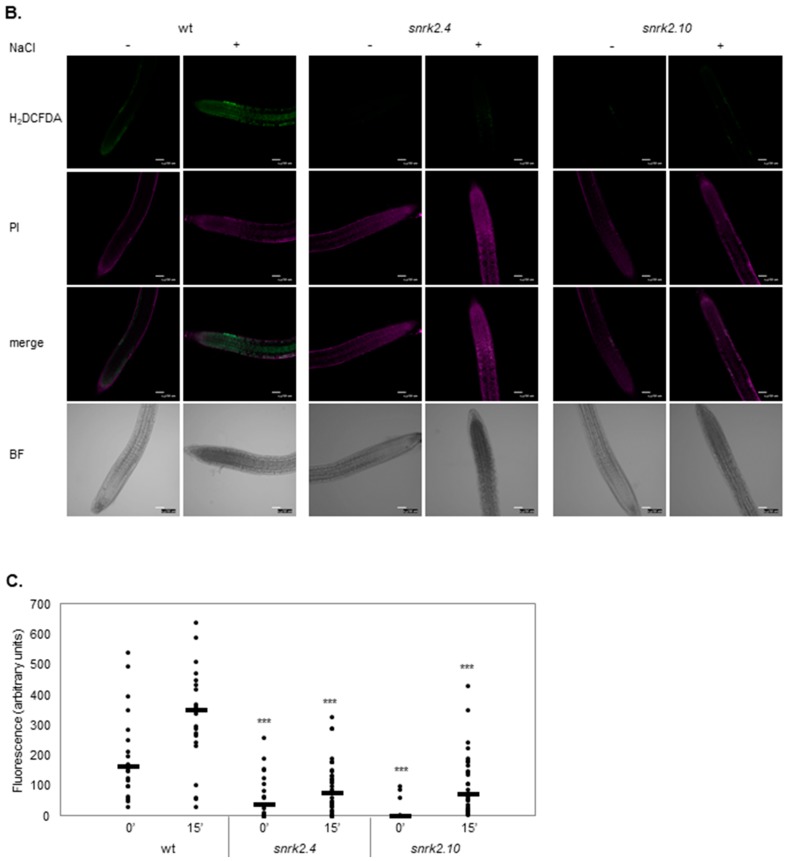
SnRK2.4 and SnRK2.10 affect ROS level in plants subjected to salt stress. (**A**). Leaves of wt plants and *snrk2.4* and *snrk2.10* mutant lines were subjected to 150 mM NaCl for the indicated time and H_2_O_2_ was determined using a luminol-based assay. Letters represent statistical differences in respect to the wt plants where a means no significant difference, and b means a significant difference [one way analysis of variance (ANOVA). Error bars represent standard deviation (SD). Three independent biological replicates, each with four samples per data point were performed. Results of all combined experiments are shown. B and C. Roots of five-day-old Arabidopsis seedlings (wt plants and *snrk2.4* and *snrk2.10* mutant lines) were stained with propidium iodide (PI; 20 µg/mL) and 2′,7′-Dichlorofluorescin diacetate (H_2_DCFDA; 30 µg/mL) and then treated for 15 min with 250 mM NaCl in ½ MS (+) or ½ MS only (−). (**B**) The production of ROS was monitored by imaging of H_2_DCFDA fluorescence in the roots using confocal microscopy; BF – bright field image, scale bars =50 µm (**C**) Fluorescence intensity of H_2_DCFDA was calculated from well-defined region of interest (4000 µm^2^) in the root meristematic zone on each single confocal section; stars represent statistically significant differences in respect to the wt plants (Mann-Whitney *U* test) where *** *p* < 0.0001; results represent data collected from at least 30 seedlings/line/conditions where each dot represents the sample value and a dash represents the median of measurements.

To check whether SnRK2.4 and/or SnRK2.10 are involved in ROS accumulation in the early response to salt stress, we compared the accumulation of H_2_O_2_ in leaves of four-week-old plants of the *snrk2.4* and *snrk2.10* knockout mutants and wild type Col-0 plants exposed to 150 mM NaCl for various time using a luminol-based assay. The mutant lines accumulated significantly less H_2_O_2_ than the wild type (wt) plants did in response to salinity (Figure 1A). The maximal level of H_2_O_2_ in Col-0 leaves was observed 30 min after the stressor application (over a three-fold increase in respect to the control level), whereas in the *snrk2.4* and *snrk2.10* mutants, the maximal H_2_O_2_ accumulation was only two-fold and occurred at 60 min. Notably, the level of H_2_O_2_ in control conditions was by ca. 30% lower in three out of four mutants tested relative to wt. The lower initial H_2_O_2_ content combined with the smaller increase resulted in the mutant plants having less than half of the H_2_O_2_ level found in the wt at the peak of the response to salt stress. Since the two independent lines of both mutants showed similar behavior for further studies, we decided to use only one line of each mutant, *snrk2.4-1* and *snrk2.10-1* (later referred to as *snrk2.4* and *snrk2.10* mutants), previously well characterized [23,39,40]. To verify the observed differences between the wt plants and the *snrk2* mutants in ROS accumulation in response to salt stress, we monitored their level in Arabidopsis roots using the fluorescent dye, dichlorofluorescin diacetate (H_2_DCFDA). We analyzed the accumulation of ROS in roots of five-day-old seedlings of wt plants, and the *snrk2.4* and *snrk2.10* mutants (Figure 1B,C) exposed to 250 mM NaCl for 15 min. Similarly to what was observed for Arabidopsis leaves exposed to the salt treatment, in roots of the *snrk2* mutant lines, the basal level of ROS was lower than in the roots of the wt plants and the ROS accumulation after the stress application was lower in those mutants in comparison with the one observed for wt seedlings. Thus, the obtained results strongly suggested the role of SnRK2.4 and SnRK2.10 in the regulation of ROS accumulation at the early stages of plant response to salinity.

### 2.2. SnRK2.4 and SnRK2.10 Regulate Expression of Genes Involved in ROS Generation in Response to Salinity

**Figure 2:**
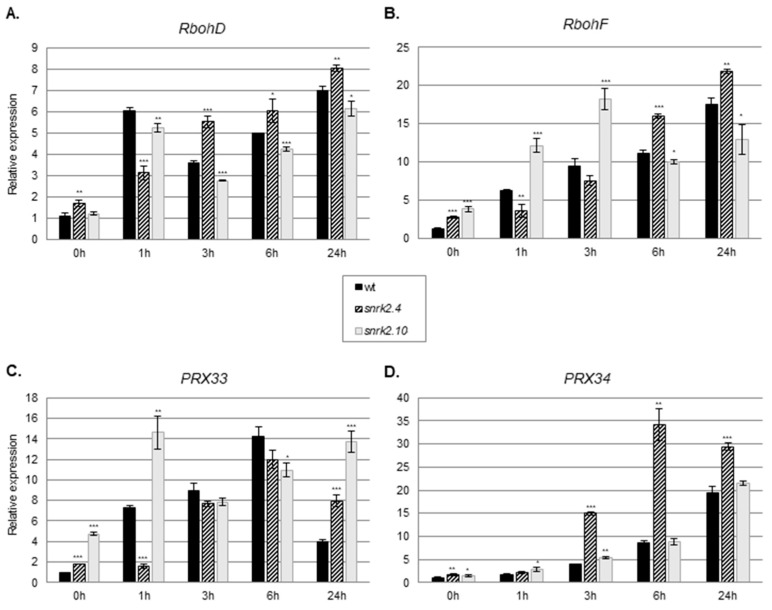
SnRK2.4 and SnRK2.10 affect the expression of genes involved in ROS homeostasis during response to salt stress. Expression (mRNA level) of (**A**). *RbohD*—*respiratory burst oxidase homolog protein D*; (**B**) *RbohF—respiratory burst oxidase homolog protein F*; (**C**) *PRX33*—*peroxidase 33*; and (**D**) *PRX34*—*peroxidase 34* was determined by RT-qPCR in wt plants and *snrk2* mutant lines subjected to treatment with 150 mM NaCl at times indicated (h); error bars represent SD; stars represent statistically significant differences in comparison with the wt plants (Student *t*-test) where * *p* < 0.05; ** *p* < 0.001; *** *p* < 0.0001. At least two independent biological replicates of the experiment were performed. Results of one representative experiment are shown.

To determine the mechanism by which SnRK2.4 and SnRK2.10 affect the H_2_O_2_ homeostasis in salt-stressed plants, we investigated their impact on the enzymes involved in ROS production and scavenging. Since mitogen-activated protein kinase (MAPK, MPK) cascades regulate the ROS homeostasis by controlling the expression of genes encoding enzymes involved in ROS production [41,42], scavenging, as well as genes involved in ROS signaling [43], we studied the expression of several genes playing a role in the regulation of ROS levels. As the first approach, we analyzed the expression of genes whose products are responsible for ROS generation. Several cellular ROS generating enzymes, especially NADPH oxidases and apoplastic peroxidases, are involved in the plant response to environmental stresses [12]. Since, in the Arabidopsis, in response to salinity ROS, are generated mainly by the RbohD and RbohF oxidases [8,44,45], we analyzed transcript levels of *RbohD* and *RbohF*. For the studies, we used two-week-old Arabidopsis seedlings of the *snrk2.4* and *snrk2.10* mutant lines and wt plants treated with 150 mM NaCl up to 24 h. At the first stage of the response to salt stress (1 h), the transcript levels of *RbohD* and *RbohF* were significantly lower in the *snrk2.4* mutant than in the wt plants (Figure 2A,B) in agreement with the lower level of H_2_O_2_ found in this mutant compared to the wt. At the later time points, this difference was reversed and the expression of *RbohD* and *RbohF* became higher in the mutant than in the wt plants. Notably, the two genes assayed showed markedly different kinetics and extents of activation during the salt treatment. The expression pattern of *RbohD* and *RbohF* in the *snrk2.10* mutant in response to the stress differed significantly from the one observed for the *snrk2.4* mutant. *RbohD* expression was hardly affected by the mutation (it was lower by only 10–15% than in the wt plants throughout the experiment), while the expression of *RbohF* was enhanced markedly at the early stages (up to 3 h) of the response and then fell to slightly below that in the wt. This behavior was opposite to that shown by the *snrk2.4* mutant.

Additionally, we analyzed the expression of genes encoding two apoplastic peroxidases (PRX), PRX33 and PRX34, known to play an important role in the oxidative burst in response to biotic stresses [46,47]. The expression of both *PRXs* was induced in the wt plants in response to salinity stress, indicating their involvement in an abiotic stress response as well (Figure 2C,D). Notably, their expression patterns were affected in a complex manner in both *snrk2* mutants indicating an involvement of SnRK2.4 and SnRK2.10 in their regulation upon salt stress. As for the two *Rboh* genes described above, also here, the two mutant lines showed contrasting responses. In the *snrk2.4* mutant, the *PRX33* gene showed a delayed induction compared to the wt, and at 24 h, its transcript level was double that in the wt. The induction of the *PRX34* gene was enhanced several fold at 3 h and 6 h and was only slightly higher at 24 h relative to the wt. In the *snrk2.10* mutant, *PRX33* showed about a two-fold higher expression level than in the wt at 1 h, nearly identical ones at 3 and 6 h, and again much higher (over three-fold) at 24 h. One should note that also in control conditions, the expression of *PRX33* was markedly up-regulated in the *snrk2.10* mutant. The *SnRK2.10* mutation had a negligible effect on the expression of the other *PRX* gene studied, *PRX34*. 

These results indicate that both kinases have an impact on the expression of *RbohD/F* and *PRX33/34* upon salt stress and that their roles are markedly different—they cannot substitute each other in this respect.

### 2.3. SnRK2.4 and SnRK2.10 Are Involved in Regulation of ROS Scavenging in Response to Salt Stress

Plants have evolved several ROS scavenging pathways, both enzymatic and non-enzymatic. We analyzed the involvement of SnRK2.4 and SnRK2.10 in the regulation of some of them upon salinity stress by comparing the responses of two-week-old *snrk2.4* and *snrk2.10* mutants and wt seedlings exposed to 150 mM NaCl.

#### 2.3.1. SnRK2s Affect CAT1 Gene Expression, Catalase Level, and Activity

**Figure 3:**
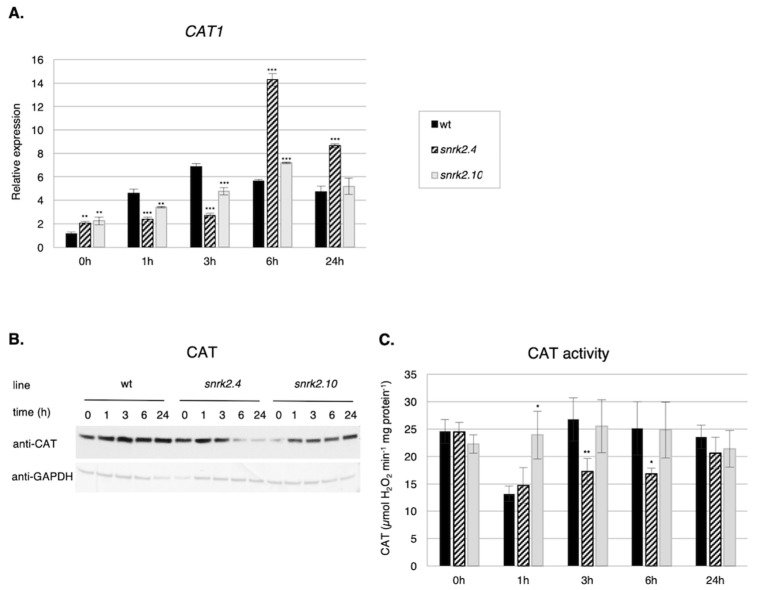
SnRK2.4 and SnRK2.10 modulate catalase (CAT) on multiple levels during response to salt stress. Wild type and *snrk2* mutants’ seedlings were subjected to treatment with 150 mM NaCl for times indicated. *CAT1* expression was determined by RT-qPCR (**A**), total catalase protein was determined by immunoblot analysis (**B**), and total catalase activity assay was performed (**C**); error bars represent SD; stars represent statistically significant differences in comparison with the wt plants (Student *t*-test) where * *p* < 0.05; ** *p* < 0.001; *** *p* < 0.0001. After exposure, membranes were stripped and reused for glyceraldehyde 3-phosphate dehydrogenase (GAPDH) detection as a loading control. At least two independent biological replicates of the experiment were performed, each with four samples per data point. Results of one representative experiment are shown.

Among the most prominent ROS scavenging enzymes are catalases. The Arabidopsis genome encodes three catalases—CAT1, CAT2, and CAT3. Expression of all of them is induced by salinity, however, the strongest changes were observed for the *CAT1* gene [48], therefore, we focused our studies on this gene. Our analysis revealed significant differences in the pattern of *CAT1* expression in response to salt stress between the *snrk2.4* and *snrk2.10* seedlings and the wt ones (Figure 3A). 

At the first stages of the response (up to 3 h), the *CAT1* transcript level was significantly lower in both mutants than in the wt, whereas at the later stages, the reverse was true, especially in *snrk2.4*. This suggests that at first, the *CAT1* expression is positively regulated by SnRK2.4 and SnRK2.10, whereas at the later stages of the stress response, the SnRK2s, especially SnRK2.4, exert an inhibitory action. Notably, the *CAT1* transcript level was up-regulated ca. two-fold in the both mutants in control conditions. In that, the effect of a lack of either SnRK2s resembled the situation observed earlier for *PRX33* and *RbohD/RbohF* genes. Unexpectedly, the differences in *CAT1* transcript levels were not reflected by the amount of catalase protein (Figure 3B) or activity (Figure 3C). The catalase protein level was in fact lower in the *snrk2* mutants exposed to salinity stress than in wt plants (Figure 3B); the lowest level was observed in the *snrk2.4* mutant after salt treatment. These data apparently indicate discrepancies between the transcript and protein levels. However, the immunoblot analysis was performed using antibodies recognizing all three isoforms of catalase—CAT1, CAT2, and CAT3. It should be mentioned that in Arabidopsis rosettes, *CAT2* and *CAT3* transcripts are much more abundant than that for *CAT1* [49,50]. Possibly the same is true for the corresponding proteins, therefore, the changes in the amount of the least abundant CAT1 were likely obscured by changes in the other two catalase isoenzymes. The latter, however, suggests that CAT2 and CAT3 could be also under the control of SnRK2s.

The catalase activity, reflecting the combined activity of the three isoenzymes, showed a different pattern for each line studied (Figure 3C); two mutants differed substantially from each other and also from the wt. Strikingly, at 1 h of salt treatment, there was a substantial drop of the catalase activity in the *snrk2.4* and the wt, whereas in the *snrk2.10* mutant, the activity basically did not change.

Taken together, our results show that during the plant response to salinity, SnRK2.4 and SnRK2.10 (especially SnRK2.4) regulate catalase at various levels, including gene expression, catalase protein level, and probably also its enzymatic activity.

#### 2.3.2. SnRK2.4 and SnRK2.10 Regulate the Ascorbate Cycle

**Figure 4:**
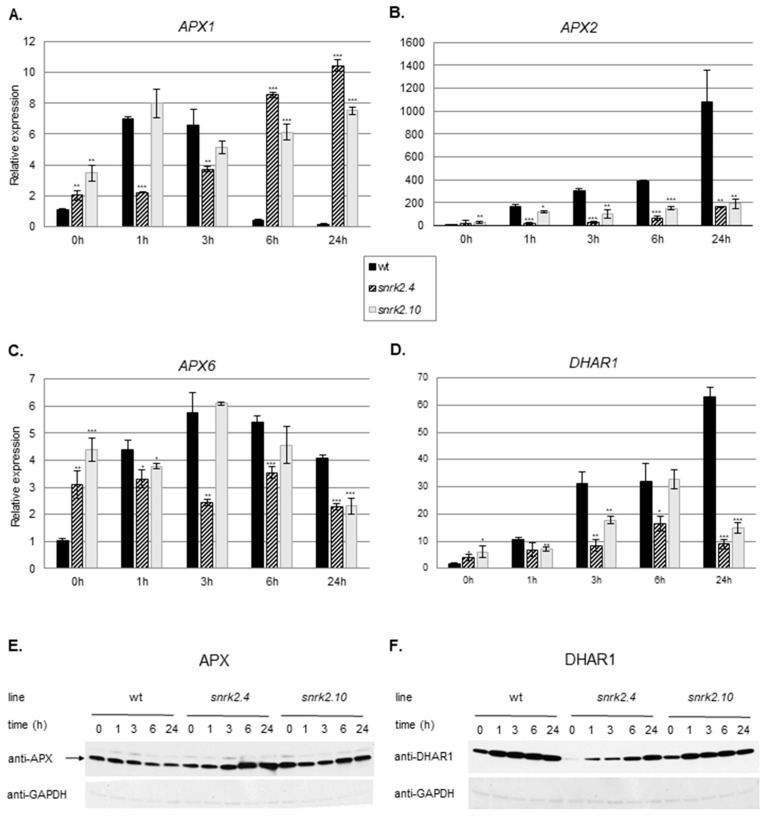
SnRK2.4 and SnRK2.10 regulate enzymes of the ascorbate cycle during response to salt stress. Wild type and *snrk2* mutant seedlings were subjected to treatment with 150 mM NaCl for times indicated. Expression of (**A**) *APX1*—*Ascorbate Peroxidase 1*, (**B**) *APX2—Ascorbate Peroxidase 2*, (**C**) *APX6*—*Ascorbate Peroxidase 6*, and (**D**) *DHAR1—Dehydroascorbate Reductase 1* was monitored by RT-qPCR; error bars represent SD; stars represent statistically significant differences in comparison with the wt plants (Student *t*-test) where * *p* < 0.05; ** *p* < 0.001; *** *p* < 0.0001. Total protein level of (**E**) APX and (**F**) DHAR1 was monitored with immunoblot analysis; after exposure, membranes were stripped and reused for GAPDH detection as a loading control. At least two independent biological replicates of the experiment were performed. Results of one representative experiment are shown.

Ascorbate is a major antioxidant in plants. To check whether SnRK2.4 and SnRK2.10 play a role in the regulation of the ascorbate cycle in plants subjected to salinity, we compared the expression of genes and their protein products involved in the ascorbate cycle (ascorbate peroxidases, APXs, and dehydroascorbate reductase 1, DHAR1) as well as the APX activity in the *snrk2.4* and *snrk2.10* mutants and wt seedlings exposed to salt stress (Figure 4A–D). Expression of all the genes encoding cytoplasmic *APXs* (*APX1*, *APX2,* and *APX6*) and *DHAR1* was highly induced in response to salinity and that response was markedly and in a complex manner affected in both *snrk2* mutants. Notably, in control conditions, their expression was significantly higher in both mutants than in the wt.

*APX1* expression was induced rapidly in response to salinity in wt seedlings, reached a maximum at 1 h and declined below control level at 6 h and 24 h, whereas in the *snrk2.4* mutant, it increased gradually between 3 h and 24 h; in *snrk2.10*, the pattern was similar except for the presence of an early (1 h) peak of expression.

A different situation was observed for the *APX2* gene. It underwent progressive very strong induction in wt plants (1000-fold increase at 24 h of salt treatment), and a similarly progressive, but much less pronounced, induction in the mutants.

*APX6* expression was rapidly induced up to six-fold in response to salinity in the wt to reach the maximum at 3 h followed by a slight drop to four-times value at time 0. In contrast, in the *snrk2.4* and *snrk2.10* mutants, where the *APX6* expression was only slightly (*snrk2.10*) or not at all (*snrk2.4*) induced upon salt application, and dropped below the initial level at 24 h in both mutants.

Immunoblot analysis (with antibodies recognizing all three APXs) showed a slight decrease of the APX amount during salt stress in the wt and substantial accumulation at the later stages (6 h and 24 h) in the *snrk2* mutants, especially in the *snrk2.4* (Figure 4E). This accumulation roughly parallels the transcript pattern of *APX1* and *APX2* and suggests that the SnRK2.4 and SnRK2.10 kinases are negative regulators of APX accumulation at the later, but not the early stages of the plant response to salinity.

*DHAR1* expression was highly and progressively induced in wt seedlings exposed to salt stress, and to a lesser extent also in the mutants. Notably, in the latter, its level fell between 6 h and 24 h of salt stress. DHAR1 protein showed strong and rapid accumulation in the wt line in response to the stress, slightly lower in the *snrk2.10* mutant, whereas in the *snrk2.4* mutant, it was barely detectable in control conditions and then grew gradually until the end of the treatment (Figure 4F), but it was significantly lower than in the wt and the *snrk2.10* mutant plants. These results suggest that the two SnRK2s, especially SnRK2.4, positively regulate DHAR1 accumulation.

The above results indicate that SnRK2.4, and to lesser extent also SnRK2.10, regulate the level of the enzymes of the ascorbate cycle during the plant response to salinity.

**Figure 5:**
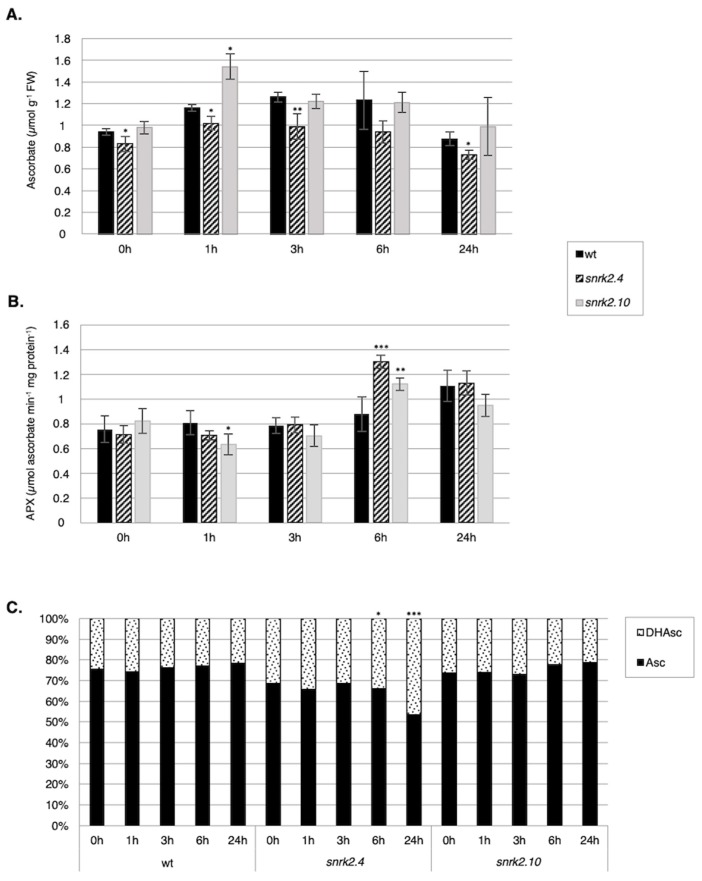
SnRK2.4 and SnRK2.10 regulate ascorbate cycle during response to salt stress. Wild type and *snrk2* mutant seedlings were subjected to treatment with 150 mM NaCl for times indicated and (**A**) ascorbate content, (**B**) ascorbate peroxidase (APX) activity, and (**C**) ascorbate redox status were monitored. Asc—ascorbate, DHAsc—dehydroascorbate; error bars represent SD; stars represent statistically significant differences from wt plants (Student *t*-test for Asc and APX activity, Chi-square test for Asc/DHAsc ratio) where * *p* < 0.05; ** *p* < 0.001; *** *p* < 0.0001. At least two independent biological replicates of the experiment were performed, each with four samples per data point. Results of one representative experiment are shown.

Next, we compared the total ascorbate (Asc) content, APX activity, and the ascorbate/dehydroascorbate (Asc/DHAsc) ratio in the *snrk2.4* and *snrk2.10* mutants and wt seedlings subjected to salinity (Figure 5). 

The total Asc content in wt plants was slightly increased in response to salt stress (from 0.9 µmol g^−1^ FW in control conditions to 1.3 µmol g^−1^ FW after 6 h of the treatment) (Figure 5A). A similar pattern was observed for the *snrk2.10* mutant, but with stronger Asc accumulation after 1 h of treatment (from 1 µmol g^−1^ FW in control conditions to 1.55 µmol g^−1^ FW after 1 h and 1.2 µmol g^−1^ FW after 3 h and 6 h of salt stress), while in the *snrk2.4* mutant, the Asc content only slightly increased in response to salt stress (0.83–1.0 µmol g^−1^ FW).

The ratio between oxidized (DHAsc) and the reduced form of Asc was virtually identical in the wt and the *snrk2.10* mutant and did not change upon salt stress (Figure 5C). In the *snrk2.4* mutant line, the fraction of the reduced form of Asc was lower than that in the other lines at all time points of the treatment and additionally showed a significant decrease after 6 h and 24 h of the stress (from 69% in control to 53% after 24 h), suggesting an increased APX activity. Indeed, measurements of the APX activity confirmed this conjecture, albeit the activity pattern did not exactly match the DHAsc/Asc pattern (Figure 5B). Generally, the APX activity reflected the APX protein level (compare Figure 4E).

## 3. Discussion

Salinity imposes ion and osmotic stresses on plant cells and leads to accumulation of ROS. The understanding of the signaling pathways controlling redox homeostasis during salt stress remains limited. A majority of the data concerning this subject pertains to the plant responses to biotic stresses. Several kinases regulating ROS production in response to pathogen infection have been identified. Some of them act as positive regulators of ROS generation via direct or indirect regulation of RbohD and/or RbohF activity or of their transcript accumulation (e.g., MPK3/6, Flagellin-sensitive 2, EF-Tu receptor, Brassinosteroid insensitive 1 associated receptor kinase 1, Botrytis-induced kinase 1, CPK5), some positively regulate PRX activity (like ZmMPK7), whereas others inhibit ROS accumulation (e.g., MPK4, AtCPK28) [51,52,53,54,55,56,57,58,59,60]. Much less is known regarding the protein kinases involved in ROS production or scavenging in response to abiotic stresses. In response to salinity, ROS are generated in different cellular compartments: Chloroplasts (by the photosynthetic electron transport), mitochondria (by the respiratory electron transport), peroxisomes, and, in the apoplast, by the action of oxidases present in the plasma membrane. The ROS generated in the various cellular compartments cross talk with each other. Recently it has been shown that, similarly to animals, the ROS-induced ROS release (RIRR) process (e.g., ROS produced in one cellular organelle or compartment induce ROS production in another one) takes place also in plants [61]. An RIRR-generated ROS wave leads to ROS amplification and signal transduction to neighboring compartments and cells. Therefore, enzymes localized to the cytoplasm or nucleus (as is in the case of the SnRK2s studied here) can indirectly affect the ROS level also in other subcellular compartments.

### 3.1. Role of SnRK2.4/SnRK2.10 in ROS Accumulation in Response to Salinity Stress

Previously published results have indicated that ABA-non-responsive SnRK2s (from group 1) regulate ROS levels in response to abiotic stresses. Diédhiou et al. [22] showed that an ABA-non-activated SnRK2, SAPK4, regulates ROS homeostasis in rice in response to salt stress, and Kulik et al. [40] that SnRK2.4 and SnRK2.10 are involved in positive regulation of H_2_O_2_ accumulation in Arabidopsis roots in the early response to heavy metal stress.

Surprisingly, studies performed on Arabidopsis, the *snrk2.2/3/6* triple, the septuple, and the decuple mutants defective in several SnRK2s did not show any correlation between SnRK2s level and H_2_O_2_ accumulation in response to osmotic stress (polyethylene glycol (PEG) treatment of Arabidopsis seedlings) [62]. However, first, the measurement was done 12 h after PEG addition. Moreover, it seems likely that individual SnRK2s might differently affect ROS production and scavenging and their defects in the multiple *snrk2* mutants could effectively cancel out, resulting in no net change in H_2_O_2_ accumulation. Finally, the role of various SnRK2s might be different in response to ABA, PEG, and salinity.

Our present studies focused on the role of two ABA-non-responsive SnRK2 kinases, SnRK2.4 and SnRK2.10, in the regulation of ROS homeostasis in Arabidopsis exposed to salinity stress. Since these kinases localize to the cytoplasm (SnRK2.10) and the cytoplasm and nucleus (SnRK2.4), we studied those enzymes involved in ROS homeostasis that in principle could be regulated by cytoplasmic and nuclear kinases: Directly by phosphorylation or indirectly at the transcriptional level. In respect to ROS production, we studied two plasma membrane NADPH oxidases, RbohD and RbohF, and two apoplastic peroxidases, PRX33 and PRX34. It has been established that RbohD and RbohF are regulated at the activity and expression levels. Their activity is tightly controlled by phosphorylation and by Ca^2+^ binding. Several kinases capable of phosphorylating RbohD/F have been identified, but the role of these phosphorylations is not fully clear. Drerup et al. [63] showed that Calcineurin B-like protein 1/9 (CBL1/9)-CIPK26 (from CBL-interacting protein kinase 26) complexes phosphorylate and activate RbohF. Similarly, Han et al. [36] presented that CIPK11 and CIPK26 phosphorylate RbohF, constituting alternative paths for RbohF activation, whereas Kimura et al. [64] suggest that the binding of CIPK26 to RbohF decreases ROS production. It has also been shown that RbohD/F are regulated also by Ca^2+^-independent kinases, like MPK8, which inhibits RbohD activity in response to wounding [65]. Some data indicate an involvement of SnRK2s in the regulation of NADPH oxidase activity. OST1/SnRK2.6, an ABA-dependent kinase, regulates the ROS level required for the stomatal closure [34]. Sirichandra et al. [35] showed that OST1 phosphorylates RbohF in vitro and suggested that this phosphorylation plays a role in its activation and possibly in the regulation of stomatal movement in response to ABA. Recently, Han et al. [36] showed that OST1 together with CIPKs is involved in RbohF activation. 

Our present results revealed that SnRK2.4 and SnRK2.10 positively regulate the ROS production at the early stages of the response to salinity; we observed significantly lower H_2_O_2_ levels in the *snrk2.4* and *snrk2.10* mutants than in wt plants salt-treated for up to 90 min. These results suggest that SnRK2.4 and SnRK2.10 might phosphorylate RbohD and/or RbohF in response to salinity and thereby regulate the ROS level. The phosphorylation of RbohD/F by SnRK2.4/10 is highly plausible since the substrate specificities of SnRK2s, CIPKs, and calcium-dependent protein kinases (CDPKs) are quite similar; all of them belong to the CDPK-SnRK superfamily [66]. The SnRK2.4 and SnRK210 kinases are activated rapidly in response to salinity, within seconds after the stressor application. Therefore, it is likely that they are involved in the earliest events of the response to salt stress, i.e., activation of the Rbohs and production of ROS responsible for triggering the defense mechanisms. However, this hypothesis needs further studies.

Our results pointed out to a role of SnRK2.4 and SnRK2.10 in the regulation of *RbohD* and *RbohF* expression. In *Arabidopsis thaliana* seedlings, the expression of *RbohD* and *RbohF* is induced in response to salinity [14,44,45,67]. At the first stages of the response (in our case, the treatment with NaCl for 1 h), the transcript levels of *RbohD* and *RbohF* were significantly lower in the *snrk2.4* mutant than in wt plants, which is in agreement with the lower level of H_2_O_2_ found in the mutants. Later, during the salt treatment, this correlation was no longer sustained and the *RbohD/F* expression in the mutant became elevated above the level observed for the wt plants. The effect of disruption of the *SnRK2.10* gene was more complex, as it has little effect on induction of *RbohD* expression, but actually enhanced that of *RbohF*.

Additionally, we analyzed the expression of genes encoding apoplastic peroxidases, *PRX33* and *PRX34*, whose involvement in the response to salt stress has not been considered so far. Expression of both *PRX*s was induced in response to salinity stress, which indicates their role in the abiotic stress response, and this induction was apparently regulated by SnRK2s. Expression of *PRX33* was significantly lower in the *snrk2.4* mutant early in the response to salinity, but became highly elevated relative to the wt plants after prolonged salt treatment. For *PRX34,* the effect of the *snrk2.4* mutation was visible only at later stages of the response and manifested as a several fold enhancement of the induction. This suggests an inhibitory role of SnRK2.4 on the salt-induced *PRX34* expression.

As for the *Rboh* genes, also here, SnRK2.10 turned out to act differently to SnRK2.4. The *snrk2.10* mutation had virtually no effect on *PRX34* expression, but greatly stimulated the expression of *PRX33* in control conditions and also at early and late response to salt stress.

**Figure 6:**
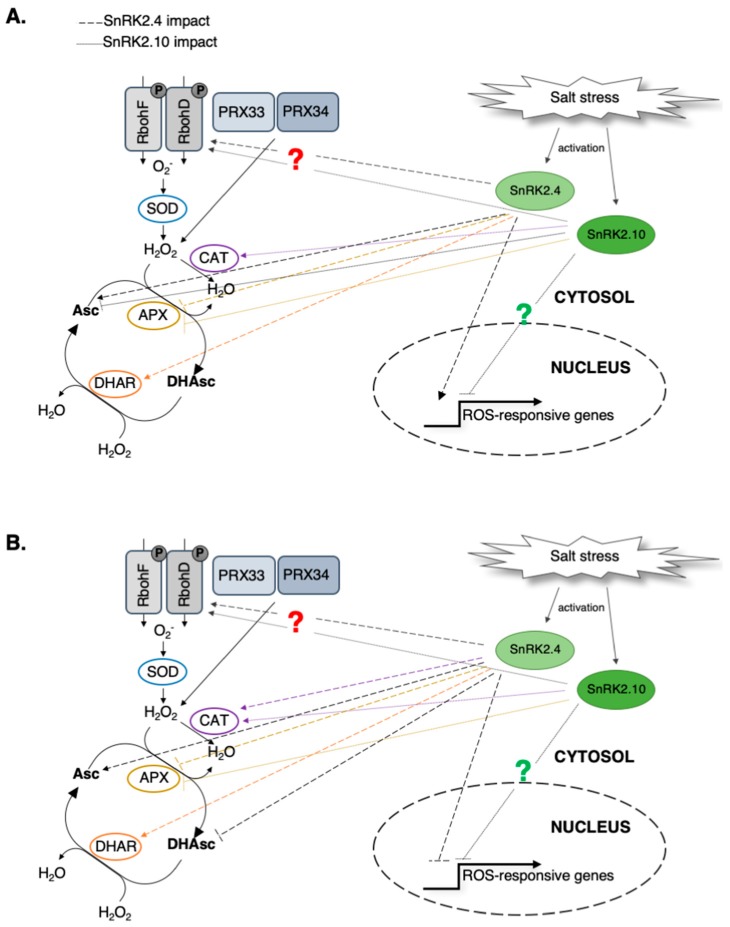
Possible roles of SnRK2.4 and SnRK2.10 in the regulation of the ROS homeostasis in Arabidopsis seedlings exposed to the salt stress. Proposed role of the SnRK2s in (**A**) early response and (**B**) late response to the salt stress. In response to salinity, SnRK2.4 along with SnRK2.10 regulate the ROS production/accumulation as well as ROS scavenging at the transcription as well as protein and/or activity levels. Detailed description in the text; dash lines—SnRK2.4 impact; dotted lines—SnRK2.10 impact; green question mark—probably indirect regulation; red question mark—plausible direct regulation by phosphorylation.

These data indicate that even though in both the *snrk2.4* and *snrk2.10* mutants, the level of H_2_O_2_ produced early in response to salt stress is significantly lower than in wt plants, the mechanisms of the regulation of ROS accumulation by the two kinases seem to be different. It should be stressed here, that unlike SnRK2.4, SnRK2.10 does not localize to the nucleus, therefore, the different modes of regulation of gene expression by these kinases are not surprising (Figure 6).

The expression of genes encoding ROS producing enzymes in the *snrk2.4* and *snrk2.10* (especially *snrk2.4*) is very different at early and late stages of the response. We propose that at the later stages the expression of genes studied might be regulated by other signaling pathways, for example, MAPK cascade(s), which are involved in controlling ROS homeostasis. Those pathways might be triggered to compensate for the low ROS level in the *snrk2s* mutants. In response to several stimuli MAPK cascade(s) control *RbohD, RbohF, PRX33*, and *PRX34* expression [52,68]. MPK3/MPK6 phosphorylate and thus activate the ERF6 transcription factor, whose targets are *RbohD* and *PRX33* in response to fungal pathogen [69,70,71]. Moreover, in *Nicotiana benthamiana* during the ETI (from effector-triggered immunity) and Elicitin-(INF1)-triggered PTI (from pattern-triggered immunity), salicylic acid induced protein kinase (SIPK, orthologue of Arabidopsis MPK6) phosphorylates four W-box binding transcription factors (WRKYs), which are responsible for the expression of *RBOHB* (ortholog of Arabidopsis *RbohD*), and positively regulates the *RBOHB* transcript level [72]. Since MPK6 and SIPK are activated in response to salinity and water deficits [37,73] and ERF6 is involved in response to the water limitation [74], it seems likely that the MPK6 pathway, and presumably some others, could overcompensate for the low expression of *RbohD* and *PRX33* at early stages of the response in the *snrk2.4* mutant.

### 3.2. Involvement of SnRK2.4/SnRK2.10 in ROS Removal under Salinity Stress Conditions

Data on the signaling pathways and protein kinases involved in the regulation of the antioxidant systems engaged in ROS scavenging in response to salinity or osmotic stress are scarce. It has been shown that GSK3 kinase (ASKα) regulates salt stress tolerance of Arabidopsis by phosphorylation and activation of glucose-6-phosphate dehydrogenase, an enzyme important for maintaining the cellular redox balance [75]. Zong et al. [76] have shown that ectopic expression of *ZmMPK7* in *Nicotiana tabaccum* enhances peroxidase activity, which results in lower accumulation of H_2_O_2_ in response to osmotic stress. It has been reported that several protein kinases of MAPK cascades as well as CIPK are involved in the regulation of expression and/or activity of CAT1 [43,48,77].

We analyzed here the impact of SnRK2.4 and SnRK2.10 on several enzymes involved in ROS scavenging (CATs, APXs, and DHAR1). A comparison of the changes in the *CAT1* transcript level in the *snrk2.4*, *snrk2.10*, and wt plants exposed to salinity indicated that SnRK2.4, and to a lesser extent also SnRK2.10, positively regulate the expression of *CAT1* during the first stages of the stress response. However, similar to what was observed for *RbohD*, at the later stages of the response, the impact of the *snrk2* mutations became just the opposite, which results in a higher *CAT1* expression than in the wt plants. We conjecture that this effect was not due to a direct regulation of *CAT1* expression by SnRK2.4/SnRK2.10, but rather because of reflected activation of some other signaling pathway(s) in order to compensate for the low *CAT1* expression. One such pathway might be again the MPK6 cascade, known to mediate *CAT1* expression and H_2_O_2_ production [77].

Besides the regulation of the *CAT1* expression, SnRK2.4 and, less markedly, also SnRK2.10 positively regulated catalase protein accumulation and activity during salt stress, as they were both significantly lower in the *snrk2* mutants exposed to the stress than in the wt. The discrepancy between the enhanced expression of *CAT1* later in the response and the lower catalase protein level and activity indicates that in response to salinity, the SnRK2s affect not only CAT1, but most likely also the CAT2 and CAT3 levels, in opposing directions. Since CAT2 and CAT3 are more abundant than CAT1 in Arabidopsis seedlings, it is likely that the catalase activity, which we measured, represented mainly the activity of CAT2 and CAT3. It is plausible that SnRK2s regulate also the expression of *CAT2* and/or *CAT3* genes. We observed lower catalase activity in the *snrk2.4* mutant exposed for up to 6 h to the salt stress. It is not clear whether SnRK2s modulate the enzyme(s) specific activity or only the catalases’ protein level or through the phosphorylation they impact the targeting of catalases into the peroxisomes (their final destination). Phosphorylation of catalases by SnRK2s is quite feasible, since it has been shown that salt overly sensitive 2 (SOS2), a kinase belonging to the SnRK3 subfamily, interacts with CAT2 and CAT3 and possibly influences H_2_O_2_ accumulation in response to salinity [78]. SOS2 localizes to the plasma membrane and cytoplasm and its substrate specificity is nearly the same as that of the SnRK2’s.

The available information on the regulation of the ascorbate cycle in response to abiotic stresses and the protein kinases is very limited. It has been suggested that in response to strong light, the SnRK2.6/OST1 kinase activates *APX2* expression [79]. Pitzschke and Hirt [80] have speculated that MAPK cascades could be involved in ascorbate cycle regulation based on transcriptomic analysis performed on *mapk* mutants and wt plants. They revealed that several genes encoding enzymes involved in ascorbate biosynthesis and metabolism were differentially expressed compared to wt plants [43], but those results have not been confirmed so far.

Our data regarding the expression of genes encoding selected enzymes of the ascorbate cycle, APX (APX1, 2, 6) and DHAR1, indicate that SnRK2.4 and SnRK2.10 also modulate the ascorbate cycle. The two kinases had a significant impact on *APX*s’ expression upon salt stress, but the different *APX* genes were regulated differently. The effect of the *snrk2* mutations on the *APX1* expression profile was similar to those observed for *RbohD* or *PRX33*. At present, we do not know how the *APX2* and *APX6* up-regulation by SnRK2s affects the overall APX activity. The combined APX protein level in the mutants and wt plants exposed to the stress correlated well with the level of expression of *APX1*, but not of *APX2* or *APX6*. These data indicate that most likely APX1 has the largest share in the overall cytoplasmic APX pool, and importantly, SnRK2.4 and SnRK2.10 play a role in its regulation. Furthermore, another enzyme of the ascorbate cycle that regenerates DHAsc to Asc, DHAR1, was strongly up-regulated at both the transcript and protein levels by SnRK2.4 and slightly less by SnRK2.10 in plants exposed to salinity.

The total Asc level and the Asc/DHAsc ratio were significantly lower in the *snrk2.4* mutant in comparison with the wt plants in response to salinity, which suggests that SnRK2.4 kinase positively regulates Asc accumulation. In agreement with these data, the APX activity was higher in *snrk2.4* than in the two other lines studied. Taken together, these data indicate that SnRK2.4 plays a substantial role in the regulation of the ascorbate cycle in response to salt stress, by direct or indirect regulation of APX and DHAR1 (Figure 6).

Discussing our results, one issue should be pointed out—the role of the circadian clock in the regulation of the redox homeostasis. Circadian clocks regulate the plant growth and development as well as responses to multiple environmental cues, both biotic as well as abiotic [81,82]. Numerous genes involved in the response to osmotic stress and ABA signaling have been identified as circadian clock-dependent, including *SnRK2.6* and several genes encoding stress-responsive transcription factors (for review see [83]). It has been shown that the redox homeostasis (ROS production, scavenging, and expression of ROS-responsive genes) is tuned with diurnal and circadian rhythms, for example, the expression of *CAT1* and *CAT3* is highest at noon, whereas *CAT2* is highest at dawn [84]. On the other hand, in the feedback response, ROS signals affect clock responses [84,85]. Since our results show that SnRK2.4 and SnRK2.10 regulate the ROS level, it is highly likely that the kinases have some impact on the circadian clock. We also conjecture that expression of SnRK2.4 and/or SnRK2.10 might be regulated by a circadian rhythm. It should be stressed at this point that the expression of some genes studied by us might be affected not only by the salt, but also, to some extent, by the diurnal/circadian rhythms. 

It has been reported that the transcriptomic analysis using a circadian-guided network approach might be used for identification of the genes involved in the early sensing of mild drought [86], indicating again a close relation between the stress responses and the circadian clock. Salt stress and dehydration signaling pathways have several common elements, including SnRK2s. Importantly, dehydration accompanies salt stress and it has been shown that not only ABA-activated SnRK2s, but also SnRK2, which are not activated in response to ABA, e.g., SnRK2.10 are involved in the plant response to a water deficit [39]. Therefore, when analyzing the plant response to salinity stress, as well as all other stresses, one should be aware of circadian/diurnal rhythms, which play a role in tuning those responses [84,85,86].

Regulation of the plant response to salt stress by SnRK2s is complex, and our knowledge on this subject is very limited. To provide the full picture, presenting the role of SnRK2s in the salt stress response, additional extensive work is required, e.g., the elucidation of the interplay between SnRK2s, ROS, circadian clock, and various signaling pathways.

**Figure 7:**
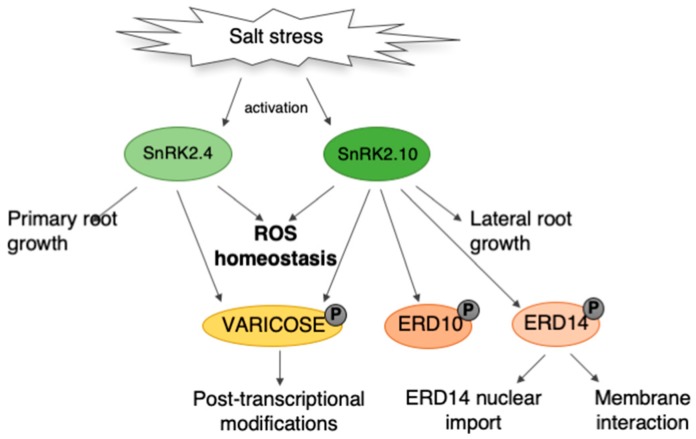
Schematic model illustrating the role of SnRK2.4 and SnRK2.10 in Arabidopsis’ response to salt stress. SnRK2.4 and SnRK2.10 modulate root growth under the salinity conditions. Moreover, in response to salt stress, the ABA-non-activated SnRK2s phosphorylate VARICOSE (VCS), a protein participating in mRNA decay, and two dehydrins, Early Responsive to Dehydration 10 (ERD10) and ERD14. Our results presented here revealed that SnRK2.4 and SnRK2.10 regulate the ROS homeostasis in the response to salinity.

A model summarizing our knowledge on the involvement of SnRK2.4 and SnRK2.10 in the plant response to salt stress is presented in Figure 7. In response to salinity stress, the kinases regulate root growth and architecture [23], mRNA decay (by phosphorylation of VCS) [24], have an impact on dehydrin ERD14 localization and likely interactions with plant membranes [39], and on ROS homeostasis (the results described here).

In conclusion, our data described here show that SnRK2.4 along with SnRK2.10 positively regulate the first ROS wave that transduces the salt stress signal. The kinases regulate ROS accumulation as well as ROS scavenging, by modulating the catalase level and the ascorbate cycle (Figure 6). These results suggest that the two studied SnRK2s are involved in the fine tuning of the ROS level and thus contribute to the regulation of ROS homeostasis required for the plant acclimation to unfavorable environmental conditions.

## 4. Materials and Methods

### 4.1. Plant Material, Growth, and Treatment Conditions

The following *Arabidopsis thaliana* lines were used: Arabidopsis Col-0 ecotype (“wild type”; wt); homozygous T-DNA insertion lines *snrk2.4-1* (SALK_080588), *snrk2.4-2* (SALK_146522), and *snrk2.10-1* (WiscDsLox233E9) kindly provided by Prof. C. Testerink (University of Amsterdam, The Netherlands), and *snrk2.10-2* (SAIL_698_C05) from the Nottingham Arabidopsis Stock Center (NASC). Seedlings were grown in a sterile hydroponic culture as described Kulik et al. [40] for two weeks.

For luminol-based H_2_O_2_ determination, plants were grown for four weeks on Jiffy pods (Jiffy-7, Jiffy Group) in a growth chamber under 8 h of light /16 h dark conditions at 21 °C/18 °C.

For ROS production measurements with H_2_DCFDA, Arabidopsis seedling were grown on ½ MS plates supplemented with 0.8% agar for five days in a growth chamber under 8 h of light/16 h dark conditions at 21 °C/18 °C.

Two- or four-week-old plants were treated with 150 mM NaCl for the indicated time (as described in the results section; stress was applied 2 h after the light was turn on), harvested by sieving, and immediately frozen in liquid nitrogen. Plant material was kept at −80 °C until further analysis.

### 4.2. Determination of H_2_O_2_

Luminol-based assay for H_2_O_2_ was performed according to Rasul et al. [87]. Discs of 2-mm diameter were excised from leaves of four-week-old plants using a cork borer, from 5 leaves per sample per condition, and placed into assay vials with 200 μL of MQ water, sealed with parafilm, and incubated at RT overnight. Next, stress conditions were applied (either 150 mM NaCl or MQ water as a control) and 4 μL of luminol solution [3 mM luminol dissolved in dimethyl sulfoxide (DMSO); final concentration 60 μM] was added at appropriate time points. Vials were gently mixed and luminescence was measured using a luminometer for a total time of 120 s. The measurements were performed at selected time points up to 90 min post treatment. Statistical analysis was performed using one way analysis of variance (ANOVA).

ROS detection with H_2_DCFDA was performed as described previously by Kulik et al. [40] and Srivastava et al. [88] with minor modifications. Staining of five-day-old Arabidopsis seedlings roots with PI and H_2_DCFDA was performed before treatment with 250 mM NaCl in ½ MS or ½ MS only. Single confocal sections were collected with a 20× (NA 0.75) Plan Fluor multiimmersion objective mounted on an inverted epifluorescence TE 2000E microscope (Nikon, Tokyo, Japan) coupled with an EZ-C1 confocal laser-scanning head (Nikon). H_2_DCFDA fluorescence was excited with blue light at 488 nm emitted by a Sapphire 488 nm laser (Coherent, Santa Clara, CA, USA) and detected with a 515/30-nm band-pass-filter and rendered in false green, PI fluorescence was excited with green light at 543 nm emitted by a 1 mW He-Ne laser (Melles Griot, Carlsbad, CA, USA) and detected with a 610 nm long-pass filter and rendered in false magenta. All confocal parameters (laser power, gain, etc.) and conditions were the same during the experiment. EZ-C1 FreeViewer software was used to quantify the fluorescence intensity from the 4000 µm^2^ area of the root meristematic zone in each Arabidopsis seedling. Each experimental variant was repeated at least twice with a total of 30 single images collected. Statistical analysis was performed using the Mann-Whitney *U* test.

### 4.3. RNA Extraction and RT-qPCR Analysis

Total RNA was extracted with TRI Reagent^®^ according to the manufacturer’s protocol (MRC). Approximately 150–200 mg of frozen ground plant material was used. DNA contamination was removed from the obtained RNA using a RapidOut DNA Removal kit (Thermo Scientific, Waltham, MA, USA). cDNA was synthesized from 4 μg of purified RNA using an Enhanced Avian HS RT-PCR Kit (Sigma-Aldrich, St. Louis, MO, USA) following the manufacturer’s protocol. RT-qPCR was performed on 50 ng of the cDNA using LightCycler^®^ 480 SYBR Green I Master Mix (Roche, Basel, Switzerland) and a Roche LightCycler^®^ 480 machine. Relative transcript levels were calculated according to Livak and Schmittgen [89] with *UBQ10* (AT4G05320) and *UBC* (AT5G25760) as reference genes [90,91]. Statistical analysis was performed using Student *t*-test. All primers used in this study are listed in Table S1. 

### 4.4. Protein Extraction and Immunoblot Analysis

Total proteins were extracted from frozen plant samples in two volumes of extraction buffer: 100 mM HEPES, pH7.5; 5 mM EDTA; 5 mM EGTA; 10 mM DTT; 1 mM Na_3_VO_4_; 10 mM NaF; 50 mM β-glycerophosphate; 10 mM pyridoxal 5-phosphate; 10% glycerol; and 1 × Complete protease inhibitors (EDTA-free, Roche) on a rotator for 30 min at 4 °C and then centrifuged at 12,000 rpm for 30 min at 4 °C. Protein concentration in the supernatant was measured using a Bradford Protein Assay. The extracts were used immediately or flash-frozen and kept at −80 °C for further analysis. The immunoblot blot analysis was based on a standard procedure described by Sambrook [92]. Protein samples (7–15 μg) were separated on 12% SDS-polyacrylamide gels and transferred to Immobilon^®®^ P membrane by electroblotting in transfer buffer, TB (25 mM Tris base, 192 mM glycine), overnight at 18 V. Transferred proteins were visualized by staining the membranes with Ponceau S (2% Ponceau S in 3% trichloroacetic acid). Immunodetection with anti-APX rabbit IgG (AS08 368, Agrisera, Vännäs, Sweden), anti-CAT rabbit IgG (AS09 501, Agrisera), and anti-DHAR1 rabbit IgG (AS11 1746, Agrisera) was performed as described in the manufacturer’s protocols. Anti-glyceraldehyde-3-phosphate dehydrogenase (GAPDH) rabbit IgG (raised against the CYDDIKAAIKEESEG peptide of GAPDH; BioGenes, Berlin, Germany) was used as described previously in Wawer et al. [93]. Secondary anti-rabbit antibodies (alkaline phosphatase (AP) conjugated—AS09 607, Agrisera; horseradish peroxidase (HRP) conjugated—AS09 602, Agrisera) were visualized using appropriate substrates—5-bromo-4-chloro-3-indolyl-phosphate/nitroblue tetrazolium (BCIP/NBT, Roche) for AP, and—ECL detection reagent (Pierce^TM^ ECL Western Blotting Substrate, Thermo Scientific) for HRP according to the manufacturer’s protocol. Membranes were reused for GAPDH protein detection used as a loading control for Western blots. Stripping of the membranes was performed according to Abcam online protocols.

For APX and CAT activity assays (see further), proteins were extracted from frozen plant samples (0.5 g FW) with 1 mL of ice-cold 50 mM sodium phosphate buffer, pH 7.5 or 100 mM potassium phosphate buffer, pH 7.0, respectively, containing 1 mM polyethylene glycol, 1 mM phenylmethylsulfonyl fluoride, 8% (*w*/*v*) polyvinylpolypyrolydone, and 0.01% (*v*/*v*) Triton X-100, according to Venisse et al. [94].

### 4.5. Determination of Ascorbate and Ascorbate/Dehydroascorbate Ratio

Ascorbate (Asc) and dehydroascorbate (DHAsc) was determined using a modified bipyridyl method described in detail by Polkowska-Kowalczyk et al. [95]. Statistical analysis was performed using the Student *t*-test and Chi-square test.

### 4.6. Determination of APX and CAT Activity

Ascorbate peroxidase (APX, EC 1.11.1.11) activity was assayed as described previously in Polkowska-Kowalczyk et al. [95]. Enzyme activity was expressed as µmol of oxidized ascorbate per min per mg of protein.

Catalase (CAT, EC 1.11.1.6) activity was assayed at 25 °C following the decomposition of H_2_O_2_ at 240 nm (extinction coefficient 0.036 mM^−1^ cm^−1^) according to a modified method of Aebi [96]. The reaction mixture contained 50 µL of plant extract in 1 mL 50 mM potassium phosphate buffer (pH 7.0) and 9.8 mM H_2_O_2_. Enzyme activity was expressed as µmol H_2_O_2_ decomposed per min per mg of protein.

Statistical analysis was performed using the Student *t*-test.

## Supplementary Materials

The following are available online at http://www.mdpi.com/1422-0067/20/1/143/s1, Table S1: List of primers used in this study.

## Abbreviations

APX	Ascorbate peroxidase
Asc	Ascorbate
CAT	Catalase
CDPK	Calcium-dependent protein kinase
CIPK	CBL-interacting protein kinase
DAsc	Dehydroascorbate
DHAR	Dehydroascorbate reductase
H_2_O_2_	Hydrogen peroxide
MPK	Mitogen activated protein kinase
O_2_^−^	Superoxide radical
PRX	Peroxidase
Rboh	Respiratory burst oxidase homologue
ROS	Reactive oxygen species
SnRK2	SNF-1 Related Protein Kinases type 2
