# Supplementary material for: SNF1-Related Protein Kinases SnRK2.4 and SnRK2.10 Modulate ROS Homeostasis in Plant Response to Salt Stress
Source: Int J Mol Sci. 2019 Jan 2;20(1):143. doi: 10.3390/ijms20010143 (PMC6337402; doi:10.3390/ijms20010143)
Supplement: Supplementary file 1 [file ijms-20-00143-s001.pdf]

International Journal of *Molecular Sciences*

MDPI

Table S1. List of primers used in this study.

Target gene	Gene ID	Primer name	Sequence (5′→3′)	Product size (bp)
APX1	AT1G07890	APX1qFor	GTCCATTCGGAACAATGAGG	162
		APX1qRev	CCAGTAACTTCAACGGCCAC	
APX2	AT3G09640	APX2qFor	TGGTCTTATTGCCGAGAAGC	115
		APX2qRev	GATGCCTTATCGTCCCAAAC	
APX6	AT4G32320	APX6qFor	GGCGTATTCGTTGCTTATCC	114
		APX6qRev	TGCGGAAGCATATGAGACAG	
CAT1	AT1G20630	CAT1qFor	TGTCTGCTCTGGAAATCGTG	107
		CAT1qRev	ACGAATCGTTCTTGCCTGTC	
DHAR1	AT1G19570	DAHR1qFor	GTTCAGCCAACGGGCTCTTC	111
		DAHR1qRev	CCCTTGAGGACTAATCTCCAAG	
PEX4	AT5G25760	PEX4qFor	CTGCGACTCAGGGAATCTTCTAA	60
		PEX4qRev	TTGTGCCATTGAATTGAACC	
PRX33	AT3G49110	PRX33qFor	GGTCAGGTAATCCAGTGTTGC	93
		PRX33qRev	GCTCTCCGGGGCTCAC	
PRX34	AT3G49120	PRX34qFor	CGCAGATATGCTCACCATTG	84
		PRX34qRev	TCTCTCCTTCCCAAAGGAAC	
RbohD	AT5G47910	RbohDqFor	TGATTCCAACGGCCTCTTAC	112
		RbohDqRev	GTTATTCCGGCGAGCTAATG	
RbohF	AT1G64060	RbohFqFor	CGAGATCTCCGACGATGAAAC	166
		RbohFqRev	AGCGGCGGTACTAGTAGTGG	
UBQ10	AT4G05320	UBQ10qFor	GGCCTTGTATAATCCCTGATGAATAAG	63
		UBQ10qRev	AAAGAGATAACAGGAACGGAAACATAGT	
